# The National Security Implications of Cyberbiosecurity

**DOI:** 10.3389/fbioe.2019.00051

**Published:** 2019-03-22

**Authors:** Asha M. George

**Affiliations:** Blue Ribbon Study Panel on Biodefense, Washington, DC, United States

**Keywords:** cyberbio, cyberbiosecurity, cybersecurity, biosecurity, convergence

## Abstract

The cyber- and biological sciences are converging rapidly, creating benefits, new and advantageous applications, and increasing risks to all nations. The parts of the public and private sectors that should be responsible for cyberbiosecurity are not yet sufficiently organized or supported financially. This article addresses the need to ensure that national security policy: (1) assesses cyberbiological risk and incorporates deterrent and enforcement measures; (2) sets forth clear consequences for those individuals and countries that conduct cyberbiological attacks or otherwise compromise cyberbiosecurity, without imperiling the legitimate sharing of scientific data and information; (3) establishes voluntary cyberbiosecurity standards in partnership with the private sector; (4) identifies cyberbiosecurity threats, vulnerabilities, consequences, and solutions; and (5) results from the combined efforts of all branches of government and the private sector.

## Introduction

Many fields of science depend on and are affected by the cyber revolution. The far older field of biology is no exception. In fact, the two fields of biology (the science of life and living organisms, including their physical, chemical, molecular, physiological, and developmental characteristics) and cyberology (the science, study, and theory of cyberspace and cybernetics, including communications over computer networks, Internet-connected systems and data centers, computerized systems, communications and automatic control systems in both machines, and living things) are not only interrelated, each can offer perspectives on the other, enabling greater understanding while simultaneously multiplying the possibilities for new, combined threats, previously unanticipated vulnerabilities, and unintended consequences. Murch et al. (2018) defined cyberbiosecurity as “understanding the vulnerabilities to unwanted surveillance, intrusions, and malicious and harmful activities which can occur within or at the interfaces of comingled life and medical sciences, cyber, cyber-physical, supply chain and infrastructure systems, and developing and instituting measures to prevent, protect against, mitigate, investigate, and attribute such threats as it pertains to security, competitiveness, and resilience.” Adequate cyberbiosecurity can only be achieved by taking both cyber- and biological perspectives into consideration simultaneously.

## Cyberbio Convergence

Lateral thinking intentionally connects disparate subjects to generate new ideas, products, and solutions (de Bono, 1970). Additionally, different scientific areas also converge as we gain greater understanding of their most basic, often elemental characteristics, and comprehend their similarities and sometimes, equivalence (Sharp et al., 2011). Convergence also occurs through the intentional combination of two different fields, using aspects of both to produce something new (Roco and Bainbridge, 2002).

The adjective cyberbio results from all three of these types of convergence. We laterally apply our understanding of biology to robotics, nanotechnology, data, cyberspace, cybernetics, and other cyber-related areas, just as we take our understanding of cyberology and look for the same in biology and biological systems. Organic material developed artificially and used in cyber-enabled technologies and products sometimes behaves in the same way as naturally occurring organic material (Irving, 2017). As we combine the cyber- and biological fields, we create new cyberbio threats, vulnerabilities, and consequences.

National security communities throughout the world cannot afford to ignore cyberbio convergence and the increased requirements for cyberbiosecurity associated with it. As with many scientific advancements, the challenge lies in preventing intended and unintended negative impacts on every nation (Sherden, 2011). Additionally, given the speed at which both cyber- and biological activity can occur independently, the separation between and among nations is already very small. Combined cyberbio activity could move even faster, rendering geographic separation non-existent.

Many critical infrastructure sectors can be affected, and as a result, they must play a role in assuring cyberbiosecurity. The Chemical (particularly due to the convergence of biology and chemistry), Critical Manufacturing, Defense Industrial Base, Emergency Services, Energy, Food and Agriculture, Healthcare and Public Health, and Information Technology Sectors are most affected. While some may be aware of the cyberbiological risk to their sectors, they have not yet determined how best to defend against individual cyber- and biological, let alone combined cyberbiological, risks.

Cyberbio deterrence and enforcement pose challenges for national security policymakers (Blue Ribbon Study Panel on Biodefense., 2015). It is unclear what deterrence measures can be developed or enforced in this regard, especially when deterrence and enforcement are lacking for cyber- and biological activities, individually. With regard to cybersecurity, increased support for overt counter-cyber activities and dedicated cybersecurity agencies (e.g., the governmental mitosis that first resulted in the National Security Agency and U.S. Cyber Command, and then other federal organizations, such as the Department of Homeland Security Cybersecurity and Infrastructure Security Agency, in the United States) may appear to be so large or prolific as to serve as deterrents, but it unclear how effective they will be (Nakashima, 2018). The Biological and Toxin Weapons Convention (Findlay, 2006), programs to control biological select agents (US Government Accountability Office, 2017), and laws and regulations prohibiting the use of biological material for crime, terrorism, and warfare (Hodge, 2012), create some barriers to misuse and establish some agreed upon national and international norms, but serve as imperfect deterrents in the biological arena. Deterrents and laws preventing malevolent cyberbio activity have not been legislated in many countries. Extant legislation addressing cyber- and biological risks lags behind technological advances in these fields and cannot be depended upon to address combined cyberbiological threats, vulnerabilities, and consequences.

## Consequences Without Imperiling Legitimate Information Sharing

The biological research community depends on digital systems to store and analyze data (Schatz, 2015). Of great concern are the huge amounts of data accessible via the Internet and various Cloud applications, with inadequate cybersecurity (Schneier, 2012). Intellectual property and proprietary information losses associated with digitized biological information could rise to the millions or billions, eventually resulting in economic decreases and reduced international competitiveness (Heus et al., 2017). Other national security concerns include loss of privacy, discrimination, data loss or theft, industrial and commercial sabotage, industrial hacking, exploitation of research to increase disease severity, targeting based on specific DNA patterns, and the production of dangerous and novel pathogens without physical samples (Bajema et al., 2018).

Many of the same countries that are investing large amounts in cutting-edge biological research and dual-use activities that could be used to produce biological weapons are also thought to be responsible for many of the cyber incidents with which the public and private sectors throughout the world struggle today. Advances in cyber- and biological science depend in large part on information systems and management, data storage, and the increased efficiency that computational analysis affords. Some countries may want data and information to feed their growing cyber- and biological weapons programs, increase disease and cyber-attack severity on enemy populations, target specific groups for attack, harm other economies, and boost their own economic competitiveness. Evidence of and information regarding cyberbio convergence and related products may well be the most valuable of all, allowing for the acceleration of nascent, ineffective, or slow-to-develop programs.

While we must encourage the legitimate sharing of scientific data and information, and comprehend that there are not yet reasonable or better alternatives to current cyber communications and data storage options, we must also recognize that all nations and their biological and cyberbiological research, development, science, and technology are at great risk. As a matter of national security, each country must require additional biosecurity and cybersecurity in this arena and set forth clear consequences for individuals and countries who intentionally breech whatever security measures they already utilize to obtain biological and cyberbiological data and information. We must also set forth clear consequences for individuals who do not take enough care to protect the data they generate. Increased cyberbiosecurity may make information sharing more difficult, but it will not make the legitimate sharing of data and information impossible.

## Establishment of Voluntary Cyberbiosecurity Standards

The public and private sectors agree with the need for increased cyberbiosecurity. No one is interested in losing their work to their competitors within or outside their organization, company, or country. No one is so naïve as to believe that the nobility of their efforts somehow serves as a protective shield against those who want to further their own agenda.

Considering the vast number of cyber-, biological, and cyberbiological efforts currently underway, and the inability of the private sector to protect itself against all national security threats, national governments should work with their private sectors to establish voluntary standards for cyberbiosecurity. Even if governments possess enough knowledge of the breadth and specificity of private sector research and development, they generally have few mechanisms with which to force the private sector to protect against cyberbiological threats.

There are many models for the development and implementation of standards that both the public and private sectors agree to meet (National Research Council., 2015). Fewer models exist to successfully develop incentives for meeting, and agree upon penalties for not meeting, standards. The government must work with the private sector to develop cyberbiosecurity standards, incentives, and penalties within a specified, relatively short period (e.g., 1 year). The speed at which benevolent and malevolent activity is occurring defies the protracted consensus-driven processes in which many governments, such as that of the United States, engage (The White House., 1998).

## Identification of Cyberbiological Risk and Other Solutions

While both cybersecurity and biosecurity efforts are underway (with more money and resources currently going to the former), there is an obvious gap when it comes to cyberbiosecurity. For example, even within the U.S. Department of Defense, which now possess two powerful cybersecurity organizational elements (i.e., National Security Agency, U.S. Cyber Command) as well as several organizations that conduct biological research and development using highly dangerous pathogens (e.g., U.S. Army Medical Research Institute of Infectious Diseases), efforts to ensure cyberbiosecurity are insufficient (Knapp, 2018). Governmental agencies throughout the world with responsibilities for agriculture, defense, energy, justice, labor, natural resources, and transportation address cyber- and biological threats separately. Departments of justice and other departments that investigate criminal and terrorism financing are also hobbled by weak or non-existent laws for cyberbiological and other new threats.

Some nations combine their military and intelligence activities. Others are fortunate enough to have enough resources to support both separately. In either case, military and intelligence communities throughout the world must acknowledge ongoing cyberbiological activities. These communities often lack the scientific and technological expertise needed to understand the state of science in the cyber- and biological fields, impact of their convergence, intended outcomes for investments in these areas, and how they could and do impact national security. Given the speed with which advances are occurring, intelligence communities throughout the world must assess cyberbiological capabilities, applications, and abilities to do harm. Military and other national security departments must utilize this intelligence to determine how best to protect national assets.

Each country needs a large-scale program to identify and assess cyberbiological risk. At a minimum, such a program should identify new cyberbio threats, vulnerabilities, and consequences (e.g., those associated with pathogen and biomanufacturing data systems, dual-use synthetic biology, biological intellectual property, bioeconomy). This program should result from a public-private partnership among all government agencies, and private sector companies, academic institutions, and other non-governmental organizations. Risk analysis should be rigorous, independent, critical, and comprehensive, utilizing the same or similar methodologies already developed for systems analysis.

As with all areas which are converging presently, expertise is usually very hard to come by. There are some, however, who have worked in or with both fields, who could serve as effective translators between the cyber- and biological communities. Lateral thinkers, who know how to expertly apply knowledge gained in one area to that of another to come up with new insights can also be effectively utilized. As with all relatively new threats, few experts exist now with operational expertise, but they can be developed through academic and operational training and education programs. Intelligence communities should seek to develop insiders involved in cyberbio activities. Public and private sector organizations that address futures must develop scenarios that are used to develop agricultural, diplomatic, healthcare, public health, and military requirements. Governmental and non-governmental scientists must work together to understand and address the problem, while simultaneously contributing to the cyberbio body of knowledge.

## Combined Governmental Efforts

The legislative bodies and those government agencies responsible for implementing laws must work together to reduce national cyberbiological risk.

Legislative bodies must authorize national cyberbiosecurity programs that:

- Address cyberbiological risk and incorporate deterrent and enforcement measures;
- Set forth clear consequences for individuals or countries that undertake such actions without imperiling the legitimate sharing of scientific data and information;
- Allow for the establishment of voluntary standards in partnership with the private sector;
- Identify new cyberbiosecurity threats, vulnerabilities, and consequences; and
- Develop and implement solutions.

Knowing what a government must authorize is less difficult than determining legislative jurisdiction in the cyberbio arena. It is unrealistic to expect that different elements of legislative bodies that have historically addressed either cyber- or biological risk separately will suddenly or automatically work together to develop and pass legislation that address cyberbiological risk. However, given the extremely large potential impact on each nation's bioeconomy, those legislative elements that address commerce, science, and security are best positioned to produce needed cyberbiological legislation.

Each government should also request funding in, and appropriate funding for, their budget for a national cyberbiosecurity program. Given the present cyberbiological risk to all countries, every national leader should immediately add responsibilities to reduce this risk to already funded cybersecurity and biosecurity programs and assign cyberbiosecurity oversight to a very senior-level dedicated position in their governments (e.g., the U.S. Special Assistant to the President and Senior Director for Weapons of Mass Destruction and Biodefense). Leadership should also require evaluation of cyberbiological risk to their national economies.

## Conclusion

All countries, including the United States, face risks from many sources. Collective dependence on the Internet and electronic communications, cyber- and biological contributions to national and global economies, competitive participation in the biorevolution, and new types of combinational weapons make the need to reduce cyberbiological risk both imperative and vital. We must take the opportunity afforded to us now to eliminate this transnational security gap, before it is exploited by our enemies.

## Author Contributions

The author confirms being the sole contributor of this work and has approved it for publication.

### Conflict of Interest Statement

The author was employed by the Blue Ribbon Study Panel on Biodefense.
